# A live weight–heart girth relationship for accurate dosing of east African shorthorn zebu cattle

**DOI:** 10.1007/s11250-012-0220-3

**Published:** 2012-08-25

**Authors:** Maia Lesosky, Sarah Dumas, Ilana Conradie, Ian Graham Handel, Amy Jennings, Samuel Thumbi, Phillip Toye, Barend Mark de Clare Bronsvoort

**Affiliations:** 1 Department of Medicine, University of Cape Town, Cape Town, South Africa; 2 Cornell University College of Veterinary Medicine, Ithaca, New York 14853 USA; 3 Department of Veterinary Tropical Diseases, University of Pretoria, Onderstepoort, 0110 South Africa; 4 The Roslin Institute, University of Edinburgh, Easter Bush, EH25 9RG UK; 5 Centre for Infectious Diseases, University of Edinburgh, Ashworth Laboratories, Kings Buildings, West Mains Road, Edinburgh, EH9 3JT UK; 6 The International Livestock Research Institute, PO 30709, Nairobi, 00100 Kenya

**Keywords:** East African shorthorn zebu, SHZ, Weight estimation, Cattle, Heart girth, Dosing, Kenya

## Abstract

The accurate estimation of livestock weights is important for many aspects of livestock management including nutrition, production and appropriate dosing of pharmaceuticals. Subtherapeutic dosing has been shown to accelerate pathogen resistance which can have subsequent widespread impacts. There are a number of published models for the prediction of live weight from morphometric measurements of cattle, but many of these models use measurements difficult to gather and include complicated age, size and gender stratification. In this paper, we use data from the Infectious Diseases of East Africa calf cohort study and additional data collected at local markets in western Kenya to develop a simple model based on heart girth circumference to predict live weight of east African shorthorn zebu (SHZ) cattle. SHZ cattle are widespread throughout eastern and southern Africa and are economically important multipurpose animals. We demonstrate model accuracy by splitting the data into training and validation subsets and comparing fitted and predicted values. The final model is weight^0.262^ = 0.95 + 0.022 × girth which has an *R* ^2^ value of 0.98 and 95 % prediction intervals that fall within the ±20 % body weight error band regarded as acceptable when dosing livestock. This model provides a highly reliable and accurate method for predicting weights of SHZ cattle using a single heart girth measurement which can be easily obtained with a tape measure in the field setting.

## Introduction

The accurate estimation of livestock weights is important for many purposes such as determining ration amounts, agreeing on sale prices and for ensuring the correct therapeutic dosing of animals. East African shorthorn zebu (SHZ) are multipurpose animals that serve as sources of draught power, milk and meat and contribute to household incomes throughout eastern and southern Africa (Rege et al. 2001). Milk production is largely determined by reproductive performance, which is in turn closely correlated with cow weight and body condition (Kanuya et al. 2006). Similarly, the relationship between live weight and capacity for work in SHZ used as draught animals is well established (Bartholomew et al. 1994; Fall et al. 1997). It can thus be deduced that body weight can be used to evaluate the value of an animal intended for use as breeding stock, milk production, draught power or beef. A simple, accurate method of approximating SHZ body weight in the field will thus give farmers greater bargaining authority at cattle markets, maximising the economic return on the investments made in their animals.

Livestock pathogens, such as *Trypanosoma* sp., *Babesia* sp., *Anaplasma* sp. and *Theiliera* sp., remain important constraints to livestock production in east Africa, where the main control methods available to farmers are pharmaceuticals (Perry et al. 2002; Malak et al. 2012). However, underdosing of therapeutic pharmaceuticals not only fails to control pathogens but also leads to the development of antimicrobial-resistant bacteria through selection pressure (Spellberg et al. 2008; Morgan et al. 2011). Although the risk to human health has not been clearly defined, it is a major public health concern that the resulting antimicrobial-resistant genes will be transferred to bacteria pathogenic to humans, such as *Campylobacter* spp., *Salmonella* spp. and enterotoxigenic *Escherichia coli*, and enter the food chain or environment (Shuford and Patel 2005; Mathew et al. 2007; Gousia et al. 2011; Oliver et al. 2011). This may be of even greater concern in developing countries, where antimicrobial treatment options are limited by cost and availability (Okeke et al. 2005). Though rarely reported in the literature, there is also a risk associated with overdosing, which could lead to insufficient drug withdrawal times and increased risk of meat and milk residues, in addition to being wasteful and economically inefficient. Many of these issues are cause for concern in developing countries where access to reliable estimates of weight for dosing can be difficult to obtain and where the impacts of resistance are likely to be most severely felt and least likely to be monitored or controlled.

Weighing scales, though accurate, are not commonly available nor convenient for use in an African field setting. There are many studies (Buvanendran et al. 1980; Nicholson and Sayers 1987; Nesamvuni et al. 2000; Goe et al. 2001; Abdelhadi and Babiker 2009; Ozkaya and Bozkurt 2009; Yan et al. 2009) that have aimed to estimate weights from various body measurements, but these often require several measurements per animal, which is inconvenient, time-consuming and possibly dangerous (many animals in these settings are not as familiar with handling as European counterparts). European-based weigh tapes developed for Holstein or other European beef breeds consistently overestimate the true weight of SHZ cattle, which have very different conformations (Mwacharo et al. 2006; Machila et al. 2008). It is clear that the morphologically distinct SHZ, which comprise the majority of cattle in eastern and south-central Africa, will need their own predictive model of weight, and these may need to be complex functions over the full age range. Further, it has been found (Machila et al. 2008) that farmers consistently underestimate the live bodyweight of cattle, demonstrating the need for the development of an accurate and inexpensive method. Visual estimation of live weight in many livestock species is generally regarded as very inaccurate and prone to error. This manuscript uses statistical methods to develop and validate an accurate, statistical model for SHZ cattle live weight based on heart girth measurement.

## Materials and methods

Data for the model came from two sources: a convenience sample of 241 cattle was selected at a number of livestock markets (Amukura, Kemodo, Funyula, Myanga, Ogalo, Bumala, Lugulu, Boro, Kocholya and Myanga) during June and July 2010, where some attempt was made to exclude animals with exotic genes through a seller questionnaire asking about origins and breeding, and at Mkura market in September 2010. All markets were in the Busia administrative district in a region of western Kenya near the Kenya–Uganda border. A further 462 observations were taken from the Infectious Diseases of East Africa (IDEAL) calf cohort study. Calves from 20 randomly selected sub-locations within a 45-km radius of Busia town were recruited and followed for the first 12 months of life. Each animal was observed at five weekly intervals, but to avoid issues of repeated measures, a single observation per calf was randomly drawn. The final data set therefore consisted of 703 SHZ cattle owned by smallholder farmers in western Kenya ranging in age from 1 week to fully mature. Each animal was weighed using either a pair of portable calibrated weigh beams (ZEMIC, model H8C-C3-1.5t-4B-SC) or a spring balance (for IDEAL calves up to 31 weeks of age). Their heart girth was measured using a simple measuring tape held with 1-kg tension using a light spring balance. Each animal’s sex and age (estimated via dentition in the case of market animals) were also recorded.

Statistical analysis was carried out using the R software program (R Development Core Team 2011). The data set was divided into two subsets, chosen by random selection, a modelling subset of slightly less than 50 % of the data was used to develop the statistical model and a validation set was used to validate the model. A likelihood maximised Box–Cox transformation, $h(y,\lambda) = (y^\lambda-1)/{\lambda}, \lambda \ne 0$, was used to estimate the transformation power coefficient (Box and Cox 1964). Linear regression models were then applied to the transformed data to test the significance of potential explanatory covariates. A number of models obtained from the literature were also fitted (Table 1) and compared with the model developed in this study. The validation data set was used to determine the performance of the model predictions.

**Table 1:** Model equations of form weight = given equation, breed applied to and source

Breed	Equation	Source
Fulani	1,513 − 37.97 *x* + 0.3093 *x* ^2^ + 0.000749 *x* ^3^	Buvanendran et al. (1980)
Gudali	− 438 + 4.88*x* − 0.001823 *x* ^2^	Buvanendran et al. (1980)
Boran	− 432.73 + 4.81*x*	Nicholson and Sayers (1987)
Nguni	16.58 + 0.81*x*	Nesamvuni et al. (2000)
Abyssinian SHZ	− 363 + 4.17*x*	Goe et al. (2001)
Baggara	− 92.472 + 2.4573*x* ^a^	Abdelhadi and Babiker (2009)
Holstein	− 473 + 5.21*x*	Ozkaya and Bozkurt (2009)
Brown Swiss	1,733.22− 19.84*x* + 0.07*x* ^2^	Ozkaya and Bozkurt (2009)
Crossbred	− 935 + 7.69*x*	Ozkaya and Bozkurt (2009)
Holstein–Fresian	− 666.6 + 6.373*x*	Yan et al. (2009)
SHZ	− 409 + 4.55*x*	Kashoma et al. (2011)

Notation has been standardised so that *x* always refers to cattle heart girth

^a^The model in question used heart girth around the hump

## Results

**Fig. 1:**
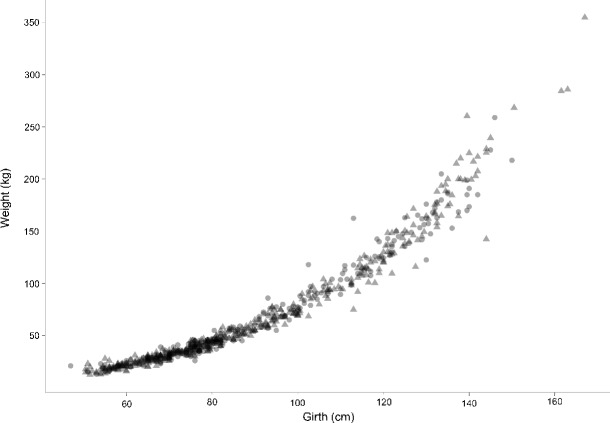
Measured heart girth versus measured weight scatter plot for 703 African shorthorn zebu cattle. The model data set is indicated by *circles* and the validation data by *triangles*

The complete data weight versus heart girth scatter plot is provided in Fig. 1, showing the modelling and validation data subsets. The modelling subset of 300 observations was used to fit the transformation and regression parameters. The Box–Cox transformation parameter, *λ*, was estimated to be 0.262. Gender, weaning status, interactions and higher order girth terms were found to be not significant once heart girth was in the model. A very simple linear model was fitted to the transformed data. The model is given as

1 $$ y_i^{0.262} = 0.95 + 0.022 x_i, $$

where *x*_*i*_ is the measured heart girth (in centimetre) for subject *i* and *y*_*i*_ is the measured weight (in kilogram) for subject *i*. This resulting model had an adjusted *R* ^2^ of 0.98 and a residual standard error of 0.08.

**Fig. 2:**
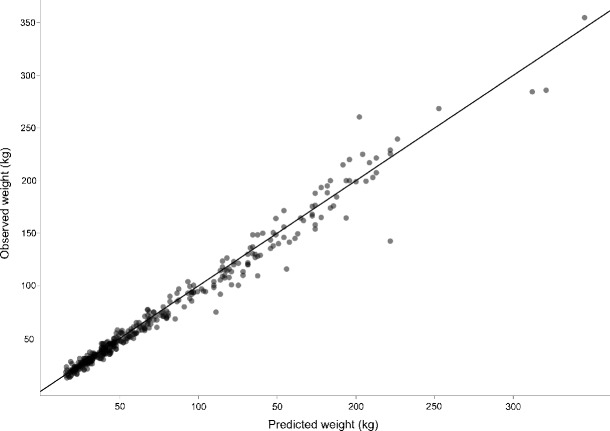
Agreement between predicted weights applying the model developed on the training data set to the validation data set and the observed weights. The line of perfect agreement is overlaid on the plot

**Fig. 3:**
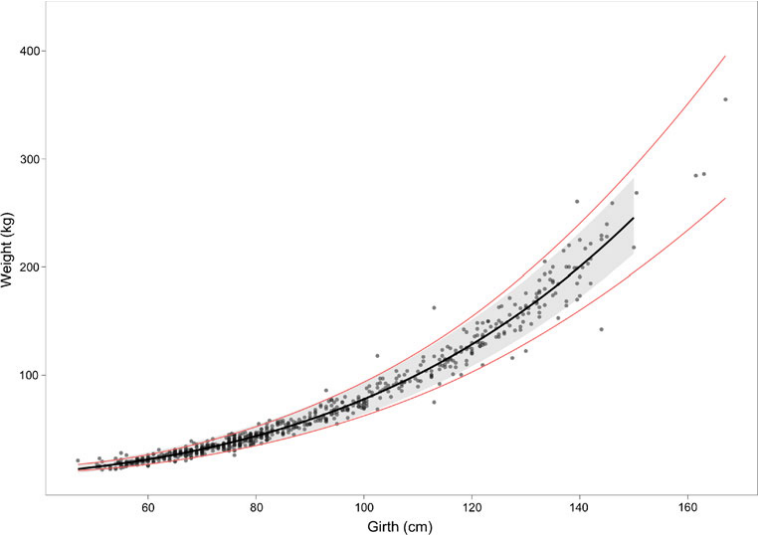
Complete data overlaid with the best fit model (*thick solid line*), 95 % prediction intervals (*grey band*) and the ±20 % body weight safe zone for dosing (*thin solid lines*). The model line and 95 % prediction intervals are those developed from the modelling data subset only

**Fig. 4:**
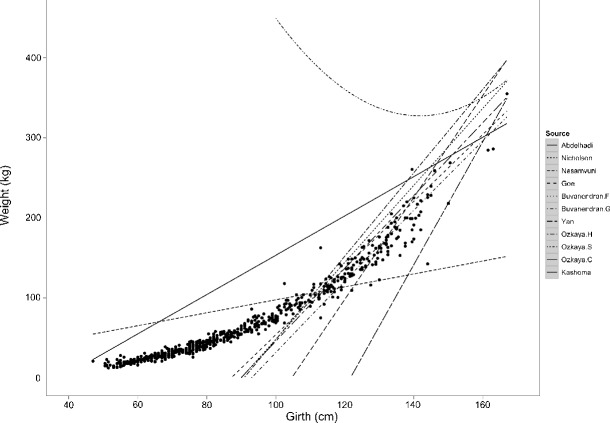
Complete data overlaid with models sourced from the literature. Model equations can be found in Table 1

The model was then used to predict weights based on heart girth measurements in the validation data set. Figure 2 shows the agreement between predicted weights and observed weights in the validation data set. 95 % prediction intervals were calculated and compared with the 20 % safe dosing zone, as established by (Machila et al. 2008), and this is shown in Fig. 3 where the model, prediction intervals and safe dosing zone have been back transformed for clarity. Figure 4 shows the result of fitting models from the literature to the complete data set; the model equations, breeds and citations can be found in Table 1.

## Discussion

The proposed model for estimation of weight via heart girth measurements satisfies statistical and practical considerations. The model is highly significant, has a very high adjusted *R* ^2^ value, shows very good performance on the validation data set and, importantly, predicts weights for which the 95 % prediction intervals fall within the safe dosing zone. Because the model does not use sex or age to stratify results, it is very amenable to transfer onto a weight tape that can be used for all ages of SHZ cattle. The potential impact of this tool for smallholder farmers throughout eastern and southern Africa is far reaching, including the accurate dosing of animals to prevent selection for antimicrobial resistance, as well as accurate estimates of slaughter weight at market, which is the primary determinant of market price (Scarpa et al. 2003). As the data presented here show a clear nonlinear relationship between heart girth and weight and the majority of the literature-sourced models are linear, it is not surprising that they were not accurate at estimating weight for this data set. It is possible that breed conformation and animal maturity also play a role in the failure of other weight estimation models to fit the data as our animals. This analysis highlights the need to have appropriate measurement tools for different breeds and the dangers of trying to extrapolate from a model into a different breed and over a different age or size range; however, it seems clear that nonlinear models must be considered. Further research is required to determine if our model can be applied to other subgroups of zebu throughout Africa.
